# Espiritualidade e Doença Cardiovascular: Valorizando a Sensibilidade Cultural na Cardiologia

**DOI:** 10.36660/abc.20250143

**Published:** 2025-04-07

**Authors:** Giancarlo Lucchetti

**Affiliations:** 1 Universidade Federal de Juiz de Fora Juiz de Fora MG Brasil Universidade Federal de Juiz de Fora, Juiz de Fora, MG – Brasil

**Keywords:** Espiritualidade, Religião, Cardiologia, Doenças Cardiovasculares, Competência Cultural

Os profissionais de saúde devem estar atentos ao fato de que seus pacientes têm valores de saúde diferentes, que podem ser influenciados por aspectos culturais e espirituais.^1^ As evidências atuais mostram que as crenças espirituais e religiosas (E/R) podem ter uma associação positiva e negativa com diferentes resultados de saúde, como depressão, ansiedade, mortalidade, qualidade de vida, hospitalização, doenças crônicas e uso de substâncias, entre outros.^2^

Neste contexto, o campo da "Espiritualidade e Saúde" tem crescido constantemente na comunidade científica, impulsionado pelo número de estudos, pelo interesse das escolas médicas e pela incorporação de diretrizes de diferentes organizações médicas.^3^

Especificamente no campo da cardiologia, estudos têm apoiado a influência das crenças E/R em fatores de risco cardiovascular (CV) (por exemplo, tabagismo, inatividade física, dieta inadequada, obesidade, diabetes, hiperlipidemia); desfechos CV (por exemplo, doença arterial coronária, insuficiência cardíaca, desfechos de cirurgia cardíaca, hipertensão); mortalidade CV; e marcadores inflamatórios biológicos.^4 , 5^ Essas evidências resultaram na incorporação da espiritualidade nas diretrizes brasileiras para hipertensão^6^ e prevenção CV.^7^

Estudos anteriores também mostraram que as crenças E/R podem ter repercussões importantes em aspectos comportamentais, como julgamentos de tomada de decisão, questões éticas e relação médico-paciente.^8^ Apesar dessas evidências, pesquisas sobre a relação entre espiritualidade e adesão ao tratamento ainda são escassas na literatura científica, conforme aponta recente revisão sistemática^9^ e um estudo prévio sobre pacientes com insuficiência cardíaca.^10^

Com o objetivo de preencher esta lacuna, um estudo recente^11^ investigou o papel da espiritualidade e da religiosidade na adesão ao tratamento entre 237 pacientes hipertensos atendidos em um hospital de ensino no Brasil. Neste estudo transversal, os autores incluíram o *Duke Religion Index* e a *Brief Multidimensional Measure of Religiousness/Spirituality* para avaliar as crenças E/R, e o *Questionnaire of Adherence to the Treatment of Hypertension* para avaliar a adesão. Os resultados do estudo revelaram que a religiosidade intrínseca e o perdão foram positivamente associados à adesão ao tratamento da hipertensão, enquanto "valores e crenças" foram negativamente associados. Os autores discutiram que os resultados positivos da religiosidade e do perdão podem ser explicados por um senso mais amplo de propósito na vida, melhor saúde mental e uma maior probabilidade de adotar comportamentos mais saudáveis (considerando o corpo como um "Templo Sagrado"). Em contraste, os resultados negativos de "valores e crenças" podem estar relacionados a concepções erradas e falta de confiança no tratamento convencional.

Esses resultados são importantes, pois trazem implicações clínicas importantes para cardiologistas e profissionais de saúde que tratam de pacientes CVs. Primeiro, esses achados destacam os "dois lados da mesma moeda" da espiritualidade na assistência médica. Se, por um lado, há uma influência positiva das crenças E/R nos resultados de saúde (acontecendo em 80% das ocasiões), por outro lado, também podem haver crenças disfuncionais que podem resultar em desfechos não favoráveis. Nesse contexto, a maneira como os indivíduos usam seus recursos de enfrentamento E/R (ou seja, a maneira como eles utilizam sua espiritualidade para lidar com seus problemas de vida e estresse) é prejudicial à sua saúde.^2 , 8^

Assim, os profissionais de saúde devem reconhecer as crenças funcionais e disfuncionais de seus pacientes, avaliando esses tópicos de forma não crítica e abordando-os ou encaminhando os pacientes para cuidados especializados.^8^ Para esse propósito, histórias espirituais podem ser utilizadas, como o FICA ou a ferramenta HOPE.^8^

Concluindo, as crenças E/R são aspectos importantes que devem ser avaliados e incorporados à prática clínica de profissionais de saúde que tratam pacientes CVs, pois podem influenciar os fatores de risco CV, os desfechos e a adesão ao tratamento. Essa visão mais ampla da doença CV pode resultar em uma medicina mais inclusiva e integrativa, valorizando a sensibilidade cultural.
