# Supplementary material for: Espiritualidade e Doença Cardiovascular: Valorizando a Sensibilidade Cultural na Cardiologia
Source: Arq Bras Cardiol. 2025 Apr 7;122(2):e20250143. [Article in Portuguese] doi: 10.36660/abc.20250143 (PMC12040385; doi:10.36660/abc.20250143)
Supplement: Translation

# Translation: Spirituality and Cardiovascular Disease: Valuing Cultural Sensitivity in Cardiology

**DOI:** 10.36660/abc.20250143i

**Authors:** Giancarlo Lucchetti

**Affiliations:** 1 Juiz de Fora MG Brazil Universidade Federal de Juiz de Fora, Juiz de Fora, MG – Brazil

**Keywords:** Spirituality, Religion, Cardiology, Cardiovascular Diseases, Cultural Competency

Health professionals should be sensitive to the fact that their patients have different health values, which could be influenced by cultural and spiritual aspects.^1^Current evidence has shown that spiritual and religious (S/R) beliefs can have both a positive and negative association with different health outcomes, such as depression, anxiety, mortality, quality of life, hospitalization, chronic illnesses, and substance use, among others.^2^

In this context, the field of "Spirituality and Health" has been constantly growing in the scientific community, driven by the number of studies, the interest of medical schools, and its incorporation into guidelines from different medical organizations.^3^

Specifically in the field of cardiology, studies have supported the influence of S/R beliefs on cardiovascular (CV) risk factors (e.g., smoking, physical inactivity, inappropriate diet, obesity, diabetes, hyperlipidemia); CV outcomes (e.g., coronary artery disease, heart failure, cardiac surgery outcomes, hypertension); CV mortality; and biological inflammatory markers.^4 , 5^This evidence has resulted in the incorporation of spirituality into the Brazilian guidelines for hypertension^6^and CV prevention.^7^

Previous studies have also shown that S/R beliefs can have important repercussions on behavioral aspects, such as decision-making judgments, ethical issues, and patient-physician relationships.^8^Despite this evidence, research on the relationship between spirituality and adherence to treatment is still scarce in the scientific literature, as noted by a recent systematic review^9^and a previous study on heart failure patients.^10^

Aiming to bridge this gap, a recent study^11^has investigated the role of spirituality and religiosity on adherence to treatment among 237 hypertensive patients attending a teaching hospital in Brazil. In this cross-sectional study, authors have included the Duke Religion Index and the Brief Multidimensional Measure of Religiousness/Spirituality to assess S/R beliefs, and the Questionnaire of Adherence to the Treatment of Hypertension to assess adherence. The study's findings revealed that intrinsic religiosity and forgiveness were positively associated with adherence to hypertension treatment, while "values and beliefs" were negatively associated. The authors discussed that the positive results of religiosity and forgiveness might be explained by a broader sense of purpose in life, better mental health, and a greater likelihood of adopting healthier behaviors (considering the body as a "Sacred Temple"). In contrast, the negative results of "values and beliefs" could be related to misconceptions and lack of trust in the conventional treatment.

These results are important since they bring important clinical implications for cardiologists and health professionals treating CV patients. First, these findings highlight the "two sides of the same coin" of spirituality in healthcare. If, on the one hand, there is a positive influence of S/R beliefs on health outcomes (happening on 80% of occasions), on the other hand, there could also be dysfunctional beliefs that could result in worse outcomes. In this context, the way individuals use their S/R coping resources (i.e., the way they rely on spirituality to handle their life problems and stress) is detrimental to their health.^2 , 8^

Hence, health professionals must recognize the functional and dysfunctional beliefs of their patients, assessing these topics in a non-judgmental manner and addressing them or referring patients to specialized care.^8^For this purpose, spiritual histories could be used, such as the FICA or the HOPE tool.^8^

In conclusion, S/R beliefs are important aspects that should be assessed and incorporated into the clinical practice of health professionals who treat CV patients since they can influence CV risk factors, outcomes, and adherence to the treatment. This broader view of CV disease could result in a more inclusive and integrative medicine, valuing cultural sensitivity.
